# Soft, Multi‐Wavelength Photoplethysmography Enables Reliable Neonatal Blood Pressure Monitoring Via Error Stratification

**DOI:** 10.1002/advs.76775

**Published:** 2026-07-27

**Authors:** Wenqi Shi, Lanlan Mi, Jiarong Chen, Biao Liu, Min Wang, Zisheng Dai, Bin Liu, Ruize Gao, Yunfeng Lin, Weiwei Guo, Guoxing Wang, Wenyi Luo, Cheng Chen

**Affiliations:** ^1^ National Key Laboratory of Advanced Micro and Nano Manufacture Technology Shanghai Jiao Tong University Shanghai China; ^2^ School of Integrated Circuits (School of Information Science and Electronic Engineering) Shanghai Jiao Tong University Shanghai China; ^3^ Department of Neonatology, Shanghai Children's Medical Center, School of Medicine Shanghai Jiao Tong University Shanghai China; ^4^ Department of Neonatology Fujian Children's Hospital (Fujian Branch of Shanghai Children's Medical Center) Fujian China; ^5^ Shenzhen Ninenovo Technology Limited Shenzhen China; ^6^ Department of Nursing, Shanghai Children's Medical Center, School of Medicine Shanghai Jiao Tong University Shanghai China

**Keywords:** deep learning, multi‐wavelength photoplethysmography, neonatal intensive care, noninvasive blood pressure, wearable device

## Abstract

Continuous noninvasive blood pressure monitoring is important for neonatal hemodynamic management. However, cuffless photoplethysmography (PPG) is still difficult to use clinically because acquisition‐related errors and their effects on blood pressure estimation are not well understood. Most studies report only overall error, which can hide error patterns under different signal‐quality and motion conditions. Here, we developed a soft multi‐wavelength wearable that combines reflective and transmissive optical paths to record complementary PPG signals. We also built NEO‐BP, a prospective clinical dataset containing synchronized multi‐wavelength PPG and invasive arterial blood pressure waveforms from 42 neonates. In a held‐out segment‐level test set without excluding samples based on signal quality (n = 9378), the green‐red‐infrared (G+R+IR) model achieved mean absolute errors of 9.67 mmHg for systolic blood pressure and 6.17 mmHg for diastolic blood pressure. Using this dataset, we established a retrospective descriptive subgroup error profile by stratifying estimation deviations across measurable acquisition and physiological conditions. Estimation errors increased under lower signal quality and stronger motion. These results identify error‐prone acquisition states, support quality‐aware interpretation of segment‐level cuffless blood pressure estimates, and provide a basis for future models that estimate the error risk of individual readings.

## Graphical abstract

A soft hybrid multi‐wavelength PPG wearable acquires neonatal signals. Synchronized PPG and invasive ABP data are segmented into fixed windows. A 1D‐EfficientNet model predicts segment‐level SBP and DBP. Model performance is examined with retrospective subgroup analysis across acquisition conditions.

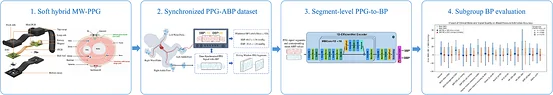

## 1 Introduction

Accurate and continuous blood pressure (BP) monitoring is critical for neonatal hemodynamic management because delayed detection can lead to complications such as intraventricular hemorrhage and ischemic organ injury [1]. However, routine neonatal care currently lacks a practical solution for continuous BP tracking. Intra‐arterial lines are invasive and therefore restricted to the most critically ill patients. Conversely, intermittent oscillometric measurements are disruptive, may cause tissue damage, and often exhibit reduced accuracy in hypotensive states [2]. These limitations create a gap in continuous BP surveillance during step‐down care, in general wards, and after discharge, motivating a truly noninvasive and continuous approach [3].

Despite the transformative potential of photoplethysmography (PPG) for cuffless BP estimation, its translation to neonatal care is stalled by fundamental physiological and data‐driven challenges. The distinct vascular characteristics of neonates, combined with a critical scarcity of high‐quality data paired with invasive gold standards, result in severe distribution shifts that undermine model generalization in real‐world neonatal intensive care unit (NICU) settings [4, 5]. Furthermore, current algorithms typically output aggregate error metrics that obscure reading‐level uncertainty. This lack of explicit reliability information prevents clinicians from distinguishing true physiological volatility from artifact‐driven noise, creating a trust gap that precludes clinical adoption [4, 5, 6].

Here, we address both barriers through a system‐level solution that integrates prospective clinical data acquisition, dataset construction, and reliability‐oriented analysis. We construct NEO‐BP by synchronizing multi‐wavelength PPG with invasive arterial pressure waveforms across eight anatomical sites [7]. Using this resource, we perform a retrospective reliability‐oriented error‐stratification analysis to characterize a descriptive subgroup error profile showing how signal quality and motion intensity are associated with estimation deviations. This profile supports quality‐aware interpretation of BP estimates, while calibrated per‐reading confidence estimation remains future work. We also validate the wearable hardware platform for sustained high‐fidelity acquisition under routine care conditions [3].

## 2 Related Work

Neonatal wearable systems have progressed rapidly with the development of soft, skin‐compatible electronics that reduce wired constraints and better align with developmental care workflows [8, 9, 10, 11]. Existing prototypes and clinical studies typically prioritize parameters such as heart rate (HR) and oxygen saturation, whereas high‐fidelity blood pressure monitoring remains limited by sensing modality constraints, waveform fidelity, battery life, and incomplete validation under heterogeneous NICU scenarios [12, 13, 14, 15].

Photoplethysmography (PPG) is a widely studied modality for cuffless BP estimation [16], but the neonatal domain introduces fundamental challenges that limit direct transfer from adult algorithms. Compared with adults, neonates exhibit distinct vascular compliance, higher heart rates, and weaker peripheral perfusion, producing a severe distribution shift that degrades generalization when models are trained on adult datasets [17, 18]. Frequent involuntary movements also introduce motion artifacts that standard denoising strategies do not fully address [19]. Meanwhile, neonatal gold‐standard datasets suitable for model training and rigorous benchmarking remain scarce. Multi‐wavelength and multi‐channel PPG approaches have been explored to exploit wavelength‐dependent penetration, spatially diverse optical paths, or channel‐wise feature fusion for BP‐related inference [5, 7]. Prior studies have addressed motion artifacts and BP estimation using different strategies. Liu et al. used a PCA‐based wearable MWPPG to reconstruct arterial and capillary pulses, derive arteriolar PTT from their phase shift, and detect motion‐contaminated segments via a PCA motion component [20]. Hou et al. used camera‐based MWPPG/rPPG, extracting G, R, and NIR waveforms to learn wavelength‐dependent morphological features with a deep model [21]. While these works affirm the value of wavelength diversity, they differ in sensing geometry, population, calibration, and motion handling. This study instead employs a soft hybrid reflective/transmissive MWPPG wearable with synchronized invasive ABP data to relate signal quality and IMU‐derived motion to BP error, and thereby evaluates wavelength combinations and reliability‐related states in neonatal NICU monitoring.

Reliability assessment has therefore emerged as a critical complement to cuffless estimation in safety‐relevant clinical workflows. Signal quality index (SQI) are widely used to detect corrupted segments, but most approaches remain limited to noise filtering or binary rejection. They rarely progress to reading‐level error predictability [22]. Aggregate metrics such as mean absolute error can also mask substantial reading‐to‐reading uncertainty in heterogeneous bedside environments. Clinicians therefore still lack a quantifiable indicator that links real‐time acquisition conditions to the probability of estimation deviation for a specific reading [4, 5]. This gap motivates reliability‐oriented analyzes that move beyond artifact suppression toward explicit, evidence‐based error characterization. Such analyzes can support future confidence modeling at the per‐reading level.

## 3 Results

### 3.1 Wearable Device Design and Characterization

To systematically evaluate the influence of PPG wavelength combinations, anatomical placement, and clinical factors on cuffless BP estimation, a wireless neonatal cuffless blood pressure (NCBP) band tailored for real‐world NICU constraints was developed (Figure 1c). The device integrates synchronized multi‐wavelength PPG and motion sensing within a hybrid reflective/transmissive optical architecture, enabling both ${\mathrm{SpO}}_{2}$ derivation and multi‐wavelength fusion. To ensure stable long‐term acquisition, the electronics are sealed in a soft, medical‐grade enclosure composed of high‐opacity black material. This optical isolation strategy effectively suppresses ambient light intrusion and minimizes internal light leakage at the skin interface, preserving signal integrity during routine care.

**FIGURE 1:**
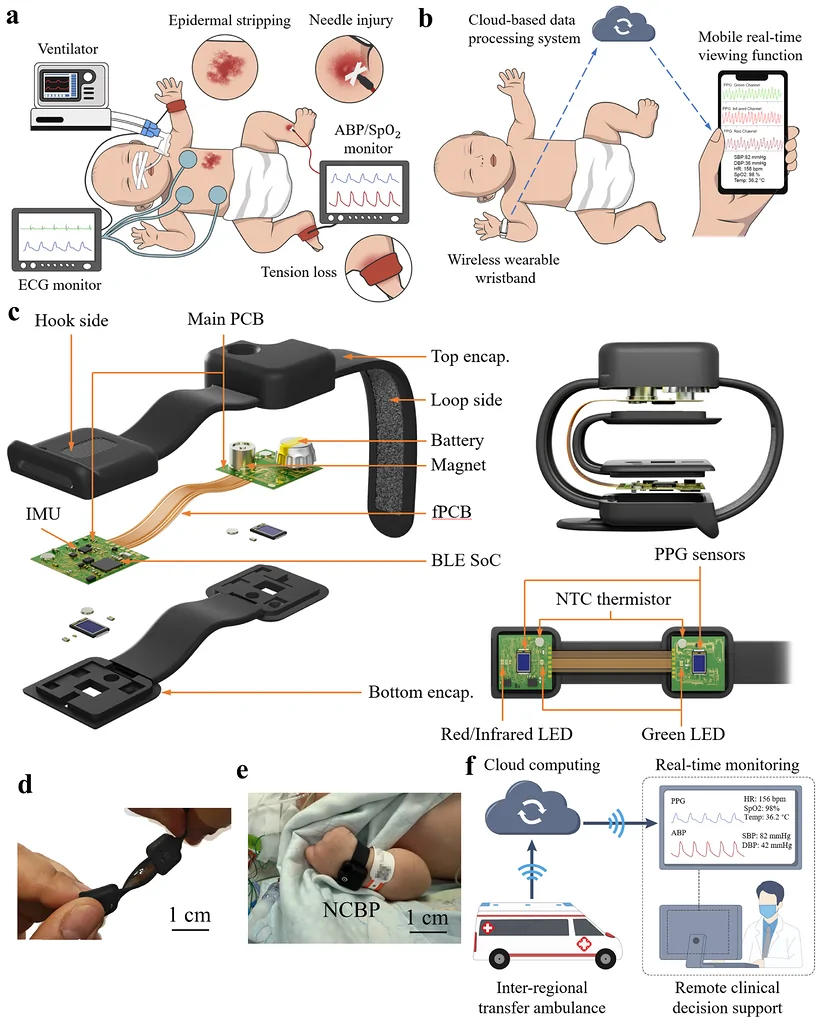
A multi‐wavelength PPG system for noninvasive blood pressure monitoring in the NICU. (a) Limitations and skin risks of traditional wired monitoring. (b) Wireless system architecture, including data acquisition, cloud processing, and mobile viewing. (c) Exploded view of the device, showing the flexible printed circuit board (fPCB), integrated components, and hybrid optical path. (d) Mechanical flexibility of the device. (e) The device worn on a neonate's wrist during data collection. (f) Application for remote monitoring during patient transfer.

The system supports real‐time wireless streaming and operates continuously for over 24 h with a 100 Hz sampling rate. Based on the 10 ms time‐division multiplexing (TDM) optical schedule and component‐level datasheet currents, the average battery‐side current was estimated to be approximately 2.5–3.6 mA, with a transient peak current of approximately 25–40 mA during light‐emitting diode (LED) pulsing and Bluetooth Low Energy (BLE) transmission. For the approximately 90 mAh single‐cell Li‐polymer battery used in the prototype, this corresponds to an expected continuous runtime of about 25–36 h, consistent with the observed >24 h operation under continuous multimodal acquisition and real‐time BLE transmission. Structural integrity under mechanical deformation is demonstrated in Figure 1d, while Figure 1e,f illustrates the clinical interface and remote viewing capability. Crucially, thermal safety was rigorously validated through a phased evaluation. Benchtop testing in a 37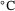 incubator confirmed negligible temperature rise under worst‐case dissipation. Subsequent clinical validation using continuous infrared thermography showed that the device surface temperature remained within 0.5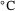 of the neonatal skin temperature after 24 h of operation (Figure 3e,f), verifying that the low‐power architecture effectively prevents localized heat accumulation.

**FIGURE 2:**
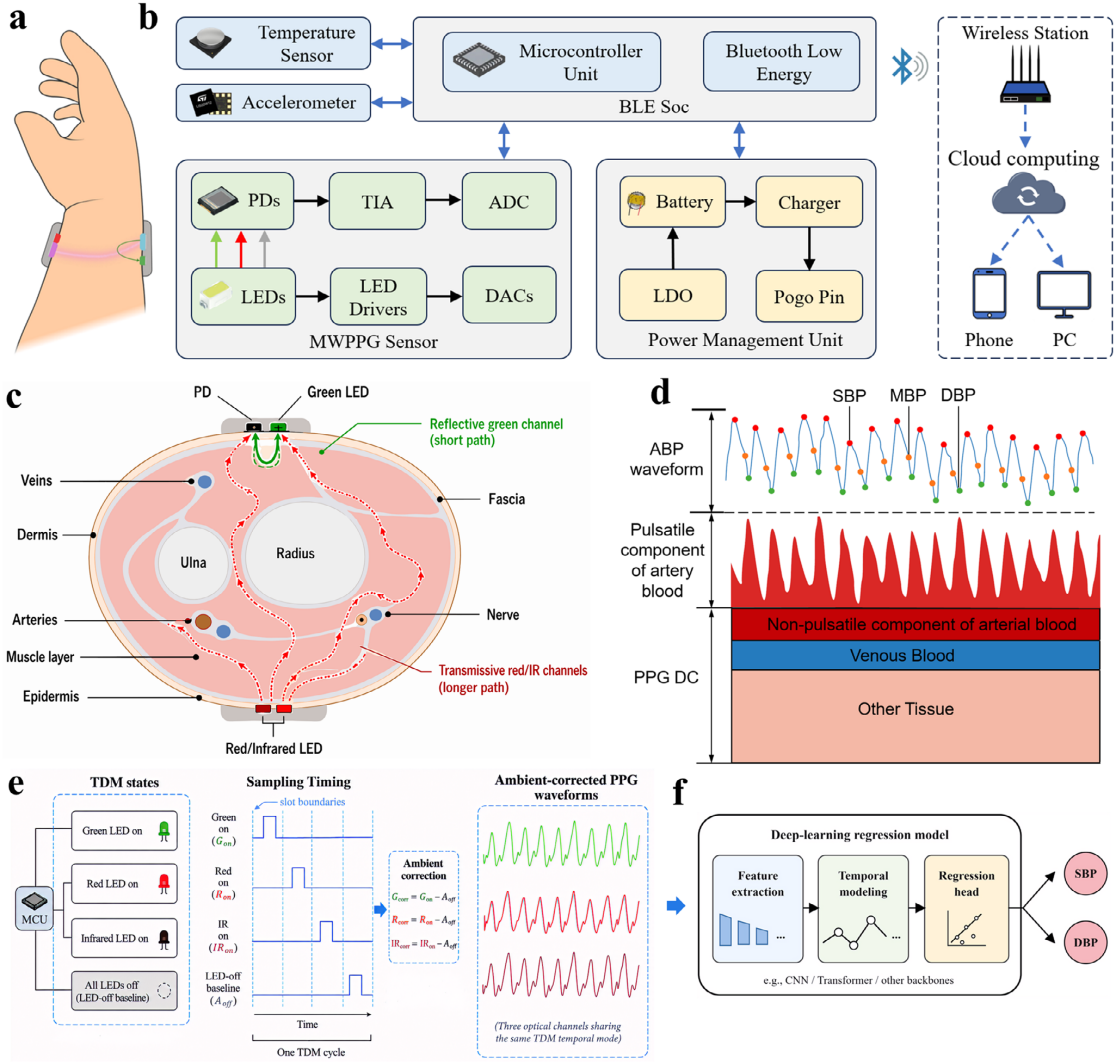
System architecture and principles for PPG‐based blood pressure measurement. (a) Device placement on the wrist. (b) System hardware block diagram. (c) Schematic optical geometry of a short‐path reflective green channel and longer‐path transillumination red/infrared channels through clinically accessible thin neonatal measurement sites. (d) Hemodynamic correlation between PPG (AC/DC components) and ABP waveforms. (e) Shared TDM timing for green, red, and infrared LEDs with LED‐off ambient‐light sampling. (f) Standard segment‐level PPG‐to‐BP regression workflow using ambient‐corrected multi‐wavelength PPG channels.

**FIGURE 3:**
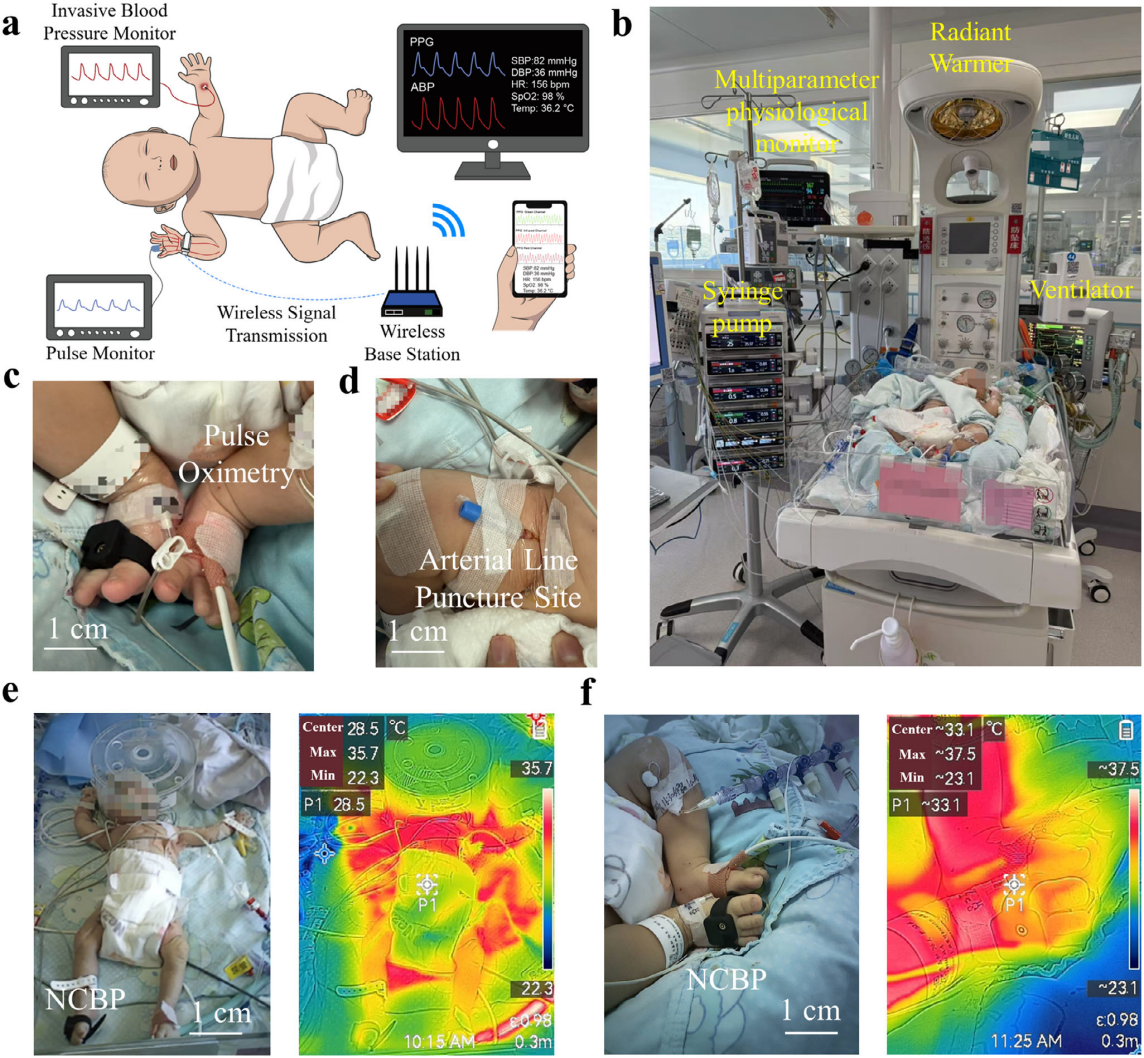
Clinical validation and data acquisition protocol in the NICU. (a) Schematic of the synchronous data acquisition setup. (b) A typical data collection scenario in the NICU. (c) Coexistence with a standard clinical ${\mathrm{SpO}}_{2}$ probe. (d) Invasive arterial catheter used as the gold‐standard reference. (e,f) Thermal safety assessment using whole‐body and local infrared imaging after 24 h of continuous wear.

### 3.2 System Architecture

This study constructed an end‐to‐end neonatal noninvasive blood pressure monitoring system, encompassing data acquisition, cloud‐based computation, and clinical presentation. The overall architecture is shown in Figure 2. At the core of the system is a wireless wearable device specifically designed for neonates, centered around a System‐on‐Chip (SoC) with integrated BLE. The SoC serves as both the microcontroller unit (MCU) and the wireless data gateway, and it centralizes data sampling, timing control, and local preprocessing.

At the signal sensing layer, the device integrates a multimodal sensor front‐end, including a multi‐wavelength PPG sensor, a high‐precision accelerometer, and a temperature sensor. During PPG signal acquisition (Figure 2a), LEDs of specific wavelengths (green: 570 nm, red: 640 nm, infrared: 950 nm) are driven to emit light. Photons penetrating the neonatal tissue are captured by a photodiode (PD). The PD converts the optical signal into a weak photocurrent, which is amplified into a voltage signal by a low‐noise transimpedance amplifier (TIA) and finally quantized into a digital waveform by a high‐resolution analog‐to‐digital converter (ADC). To ensure signal quality at the source, the firmware employs a TDM strategy to reduce optical crosstalk (Figure 2e). Within each 10 ms period, the green, red, and infrared LEDs are activated sequentially, followed by an LED‐off ambient‐light sampling phase. The LED‐off PD readout estimates the background optical/electrical baseline for that TDM frame and is subtracted from the wavelength‐specific LED‐on measurements at the system firmware layer before downstream processing, thereby suppressing ambient‐light intrusion during signal acquisition. The 10 ms TDM period provides a 100 Hz sampling rate for each PPG channel and keeps the inter‐wavelength timing offset within one frame. This setting matches the present segment‐level BP‐regression pipeline, which analyzes 8‐s PPG windows and updates every 2 s. Increasing the per‐channel rate to 400 Hz would provide denser instantaneous waveform sampling, but under the same three‐wavelength TDM structure it would also increase optical switching events, ADC readouts, wireless payload, and average power by approximately fourfold. The 10 ms period preserves timing margin for the LED‐off ambient‐light phase while supporting continuous neonatal monitoring. On the SoC, the ambient‐corrected green, red, and infrared PPG samples are timestamped, aligned, and packaged into multimodal frames for wireless transmission.

At the data computation and presentation layer, the device transmits a continuous stream of data frames wirelessly to a cloud server with low latency. The server executes a standard segment‐level PPG‐to‐BP regression pipeline used to evaluate the neonatal multi‐wavelength PPG (MWPPG) sensing system. As shown in Figure 2f, this pipeline segments the continuous ambient‐corrected MWPPG stream using a fixed‐length sliding window. This fixed‐window formulation was used to align MWPPG segments with ABP‐derived labels and to support segment‐level regression. Each windowed segment is then fed into a deep neural network model for blood pressure regression. The model automatically learns and extracts complex, nonlinear features related to blood pressure from the PPG waveforms, ultimately outputting near‐real‐time segment‐level estimates of systolic (SBP) and diastolic (DBP) blood pressure after an initial 8‐s window, with subsequent updates every 2 s. The computational feasibility of this segment‐level update pipeline is quantified together with the backbone benchmark in Section 3.6.

### 3.3 Optical Path in Tissue for Multi‐Wavelength Sensing

To acquire complementary pulsatile information under neonatal limb‐size constraints, the system's optical design employs a hybrid reflective/transmissive sensing architecture (Figure 2c). Here, the different modes refer to the spatial optical geometry rather than to differences in temporal LED operation; all wavelengths are still activated sequentially within the same TDM cycle. A reflective green channel (λ=570 nm) is positioned adjacent to the PD to acquire short‐path superficial PPG signals. In contrast, red (λ=640 nm) and infrared (λ=950 nm) LEDs are positioned opposite the PD to form longer‐path transillumination channels through clinically accessible thin neonatal measurement sites. The detected red/infrared PPG signals are interpreted as a path‐integrated pulsatile signal shaped by tissue scattering and absorption. This placement was selected to preserve the conventional red/infrared pair for ${\mathrm{SpO}}_{2}$ derivation and to provide optical‐path information complementary to the reflective green channel. The functional utility of the red/infrared channels was evaluated by ${\mathrm{SpO}}_{2}$ derivation using the red/infrared pair and by BP wavelength‐combination ablation against synchronized invasive ABP references. Hereafter, G, R, and IR denote green, red, and infrared PPG channels, respectively. In the BP ablation, R+IR outperformed the green‐only input, and G+R+IR achieved the lowest SBP/DBP errors among the tested wavelength combinations. This mixed‐geometry design allows the model to combine superficial reflective and longer‐path transmissive PPG representations. Consequently, this architecture provides complementary PPG representations compared with single‐wavelength inputs and supports the subsequent wavelength‐combination analysis.

### 3.4 Hemodynamic Correlation Between PPG Signals and Arterial Blood Pressure

The rationale for PPG‐based cuffless BP estimation relies on the intrinsic hemodynamic coupling between peripheral blood volume pulses and arterial pressure waveforms (Figure 2d). Although the PPG waveform is an optical measurement of local blood volume changes, its morphology—including slope, peak timing, and dicrotic notch characteristics—is shaped by the same cardiovascular determinants as the arterial pressure waveform, such as myocardial contractility, vascular compliance, and peripheral resistance [23, 24]. With synchronized high‐fidelity PPG and invasive ABP references, deep learning models can learn shared waveform descriptors and estimate segment‐level SBP/DBP from optical signals [25, 26, 27].

### 3.5 Prospective Clinical Study Protocol and Data Acquisition

To assess acquisition feasibility and safety in routine NICU workflows, we implemented a synchronous protocol that enables timestamp‐level alignment between wearable signals and the clinical reference (Figure 3a). Data were collected under typical NICU care settings (Figure 3b) with co‐located clinical probes such as standard ${\mathrm{SpO}}_{2}$ sensors (Figure 3c). Invasive arterial monitoring provided the gold‐standard reference waveform and derived readouts (Figure 3d).

To evaluate the system under real‐world constraints, we constructed NEO‐BP, a multimodal dataset of synchronized wearable signals and invasive gold‐standard references. The prospective NICU protocol captured high‐fidelity multi‐wavelength PPG and tri‐axial acceleration data during routine care. Recordings were obtained from eight distal anatomical sites: left/right palm, left/right wrist, left/right ankle, and left/right foot. Site effects were interpreted through acquisition‐quality variables, including PPG SQI, motion intensity, local perfusion, tissue thickness, and sensor‐skin coupling. The present analysis therefore treats site effects as acquisition‐quality effects rather than as a definitive location‐level reliability ranking. Wearable streams were timestamp‐aligned with invasive ABP waveforms and ABP‐derived monitor readouts, providing synchronized references for segment‐level BP labels rather than beat‐level waveform targets.

### 3.6 Multi‐Wavelength PPG for Cuffless BP Estimation

This study evaluates a MWPPG deep regression framework for cuffless neonatal blood pressure estimation (Figure 4a). Multi‐channel PPG segments are synchronized with invasive ABP‐derived SBP/DBP references and used to train a regressor that maps waveform morphology to segment‐level SBP and DBP. The evaluation separates signal‐level estimation from reliability characterization. The BP regressor takes ambient‐corrected, band‐pass‐filtered, and normalized MWPPG segments as the common waveform input. MWPPG fusion provides complementary optical‐path representations for the regression model. Patient identity, anatomical‐site labels, clinical‐subgroup labels, SQI, IMU‐derived motion measures, $\Delta \mathrm{HR}/\Delta {\mathrm{SpO}}_{2}$, and intra‐window BP variability are reserved for subgroup stratification or retrospective reliability analysis. This design supports subgroup error profiling and defines performance boundaries across patient‐, site‐, and condition‐related sources of acquisition heterogeneity.

**FIGURE 4:**
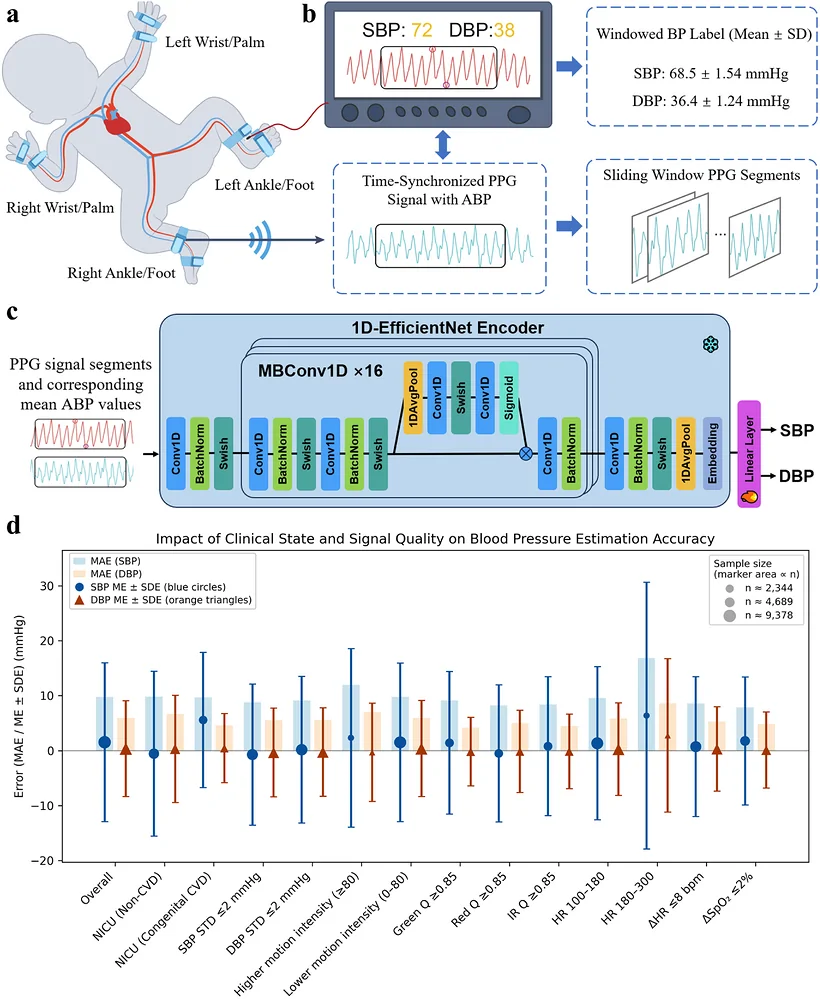
Principles and evaluation method of the multi‐wavelength blood pressure estimation algorithm. (a) The wearable device acquires raw PPG waveforms from multiple sites on the neonate's limbs. (b) Signal processing: synchronizing, segmenting, and labeling PPG data. (c) 1D‐EfficientNet model fine‐tuned for PPG‐to‐BP estimation. (d) Subgroup error profile of the best fusion model (G+R+IR): light blue and light orange bars indicate the MAE for systolic blood pressure (SBP) and diastolic blood pressure (DBP), respectively; blue circles with vertical whiskers indicate the mean error (ME) ± SDE for SBP, and orange triangles with vertical whiskers indicate the ME ± SDE for DBP. Marker area is proportional to subgroup sample size (n).

To evaluate the method's effectiveness, a performance comparison of different PPG signal combinations was conducted on the held‐out testing set without SQI‐based screening (Table 4). Because the BP regressor used wavelength‐specific PPG waveforms rather than ${\mathrm{SpO}}_{2}$ ratio‐of‐ratios features, this ablation directly evaluated BP‐relevant information in each optical channel. All configurations used the same neonatal test set, preprocessing pipeline, ABP‐derived labels, and regression backbone. The R+IR configuration served as a green‐free red/infrared ablation and achieved SBP/DBP MAEs of 11.59/6.92 mmHg, compared with 18.67/9.73 mmHg for green‐only input. Adding red and/or infrared channels to the green baseline also reduced SBP/DBP MAE: G+R, G+IR, and G+R+IR achieved 10.10/6.62, 10.54/6.90, and 9.67/6.17 mmHg, respectively. G+R+IR achieved the lowest SBP and DBP MAEs and SDEs among tested wavelength configurations. These results support the practical value of MWPPG in this neonatal setting and indicate that red/infrared PPG provides both green‐independent and green‐complementary BP‐related information. To contextualize the regression backbone within the same waveform‐level input paradigm, we further benchmarked one‐dimensional EfficientNet (1D‐EfficientNet) against representative temporal deep‐learning backbones using the same held‐out test subset, preprocessing pipeline, MWPPG inputs, ABP‐derived labels, and training/evaluation protocol. As summarized in Table 1, 1D‐EfficientNet achieved the lowest mean BP MAE, SBP MAE, and DBP MAE among the tested temporal backbones, with SBP/DBP MAEs of 9.67/6.17 mmHg. Following the PPG‐BP benchmark of González et al. [7], the mean baseline used the training‐set mean SBP and DBP as constant predictions for all held‐out test segments. This baseline yielded a BP mean MAE of 12.92 mmHg, with SBP/DBP MAEs of 16.88/8.97 mmHg and SBP/DBP SDEs of 20.77/12.99 mmHg. These results support the selection of 1D‐EfficientNet as the primary regression backbone for the present MWPPG‐based neonatal BP estimation pipeline.

**TABLE 1:** Performance comparison of representative temporal backbones using MWPPG. BP mean MAE was calculated as the average of SBP MAE and DBP MAE.

Model	BP mean MAE	SBP MAE	SBP SDE	DBP MAE	DBP SDE
1D‐EfficientNet	7.92	9.67	13.79	6.17	9.58
Informer	8.37	10.04	14.69	6.69	10.17
ResNet1DMoE	8.89	10.48	15.27	7.29	10.71
PatchTST	8.95	10.68	15.60	7.22	10.85
CSFM‐base	9.13	11.13	16.03	7.12	10.82
Mean baseline	12.92	16.88	20.77	8.97	12.99

*Note*: BP mean MAE was calculated as the average of SBP MAE and DBP MAE. MAE and SDE are reported in mmHg. The mean baseline used the training‐set mean SBP and DBP as constant predictions for all test segments.

**TABLE 2:** Computational efficiency comparison of representative temporal backbones under the same inference benchmark.

Model	MACs (M)	Params (M)	Latency (ms)	Throughput (samp./s)	Size (MB)	Memory (MB)	Power (W)	Energy (J/samp.)
1D‐EfficientNet	211.800	3.318	6.610	151.390	12.790	22.530	1.800	0.012
Informer	1336.380	4.013	5.640	177.400	16.100	49.330	3.640	0.021
ResNet1DMoE	52.770	6.318	11.100	90.110	24.150	35.500	3.320	0.037
PatchTST	268.880	3.460	1.640	610.000	13.200	23.030	3.520	0.006
CSFM‐base	8429.820	108.573	8.570	116.740	414.170	428.370	1.320	0.011

*Note*: Latency, throughput, processor‐side power, and energy per sample characterize neural‐network inference in the benchmark environment, rather than wearable‐device battery consumption.

**TABLE 3:** Detailed performance metrics for blood pressure estimation across all subgroups.

Subgroup	n	Systolic Blood Pressure (SBP)			Diastolic Blood Pressure (DBP)		
		MAE	ME	SDE	MAE	ME	SDE
Overall	9378	9.67	1.39	13.79	6.17	0.60	9.58
NICU (Non‐CVD)	6581	10.20	0.03	14.76	6.84	0.59	10.67
NICU (Congenital CVD)	2797	8.42	4.58	10.53	4.59	0.62	6.29
SBP STD ≤ 2 mmHg	7602	8.97	−0.22	12.75	5.65	−0.12	8.59
DBP STD ≤ 2 mmHg	8234	9.07	0.28	12.89	5.65	−0.13	8.58
Higher motion intensity (≥80)	69	14.23	−1.32	19.01	11.08	0.52	16.37
Lower motion intensity (<80)	9309	9.63	1.39	13.75	6.14	0.60	9.53
Green Q ≥ 0.85	3267	9.09	1.40	12.97	4.22	−0.19	6.24
Red Q ≥ 0.85	3103	8.22	−0.52	12.48	4.98	−0.16	7.47
IR Q ≥ 0.85	3255	8.36	0.79	12.65	4.45	−0.15	6.78
HR 100–180 bpm	8411	9.54	1.33	13.94	5.85	0.25	8.44
HR 180–300 bpm	205	16.83	6.37	24.26	8.63	2.76	13.95
ΔHR≤ 8 bpm	6879	8.57	0.71	12.73	5.24	0.30	7.67
$\Delta {\mathrm{SpO}}_{2}\leq$ 2%	4866	7.88	1.75	11.65	4.80	0.09	6.93

**TABLE 4:** Controlled wavelength‐combination ablation for blood pressure and heart‐rate estimation across different PPG wavelength inputs. Metrics include MAE (mean absolute error), ME (mean error), SDE (standard deviation of error), CPE (clinical prediction error within x mmHg), and RE (relative error). All wavelength combinations were evaluated using the same preprocessing pipeline, ABP‐derived labels, and regression backbone; R+IR denotes the green‐free red/infrared ablation.

Parameter	Metric	Unit	Green	Red	Infrared	G+R	G+IR	R+IR	G+R+IR
SBP	MAE	(mmHg)	18.67	19.81	17.47	10.10	10.54	11.59	9.67
	ME	(mmHg)	14.59	−2.18	1.01	1.78	2.22	2.82	1.39
	SDE	(mmHg)	19.82	25.00	21.51	14.44	15.13	16.09	13.79
	CPE5	(%)	19.89	14.96	15.95	39.36	38.12	36.69	40.82
	CPE10	(%)	37.26	30.86	31.87	64.06	62.91	57.58	64.83
	CPE15	(%)	50.63	44.98	45.98	78.01	77.63	70.73	78.95
DBP	MAE	(mmHg)	9.73	11.12	9.80	6.62	6.90	6.92	6.17
	ME	(mmHg)	6.12	3.25	4.86	0.99	1.26	1.10	0.60
	SDE	(mmHg)	12.87	15.37	13.34	10.22	10.56	10.77	9.58
	CPE5	(%)	40.00	34.70	35.69	55.73	54.96	54.82	59.43
	CPE10	(%)	66.94	59.23	65.31	80.43	79.10	78.35	82.17
	CPE15	(%)	80.82	76.68	83.90	90.13	89.09	89.35	91.17
HR	MAE	(bpm)	12.46	20.98	14.65	5.40	5.77	6.30	5.32
	RE	(%)	8.34	14.54	10.19	3.72	3.93	4.35	3.68

To further evaluate computational feasibility, we profiled the tested temporal backbones under the same inference benchmark (Table 2). PatchTST achieved the lowest latency and energy per sample but showed higher BP error than 1D‐EfficientNet. 1D‐EfficientNet therefore provided the best accuracy–efficiency balance, combining the lowest BP mean MAE with a 6.61‐ms per‐segment latency, 151.39 samples/s throughput, a 12.79‐MB model size, and a 22.53‐MB memory footprint. This latency was far below the 2‐s update interval, supporting near‐real‐time segment‐level BP updates with the selected backbone.

After confirming the advantage of the fusion model, a systematic subgroup analysis was conducted to investigate its performance boundaries in a clinical setting. Figure 4d visualizes the analysis results for a selection of typical subgroups to illustrate how the estimation error varies under different clinical and signal‐related conditions. Each subgroup in the figure includes metrics such as sample size (n, represented by the marker area), MAE (represented by bars), and ME ± SDE (represented by the error markers). The specific numerical values for these visualized subgroups, along with detailed performance metrics for all subgroups, are listed in Table 3.

To complement the aggregate subgroup profile, Figure 5 provides representative examples of BP‐estimation performance across three conditions: temporal trend agreement, signal quality, and motion artifacts. Panel a compares estimated and reference SBP/DBP over consecutive windows from two recordings. Panel b pairs high‐ and low‐quality 8‐s PPG windows (top) with their local BP trends (bottom); a gray marker indicates each window's position in the trend. Panel c shows a motion‐contaminated segment, including model‐input PPG, original PPG, baseline‐corrected acceleration magnitude, and the corresponding BP trend. MAE and SDE quantify local error magnitude and dispersion over each segment; Green‐Q, Red‐Q, and IR‐Q denote channel‐specific SQI values for the selected window. These illustrative examples are from the trained waveform‐only G+R+IR model and do not alter model inputs or exclusion criteria.

**FIGURE 5:**
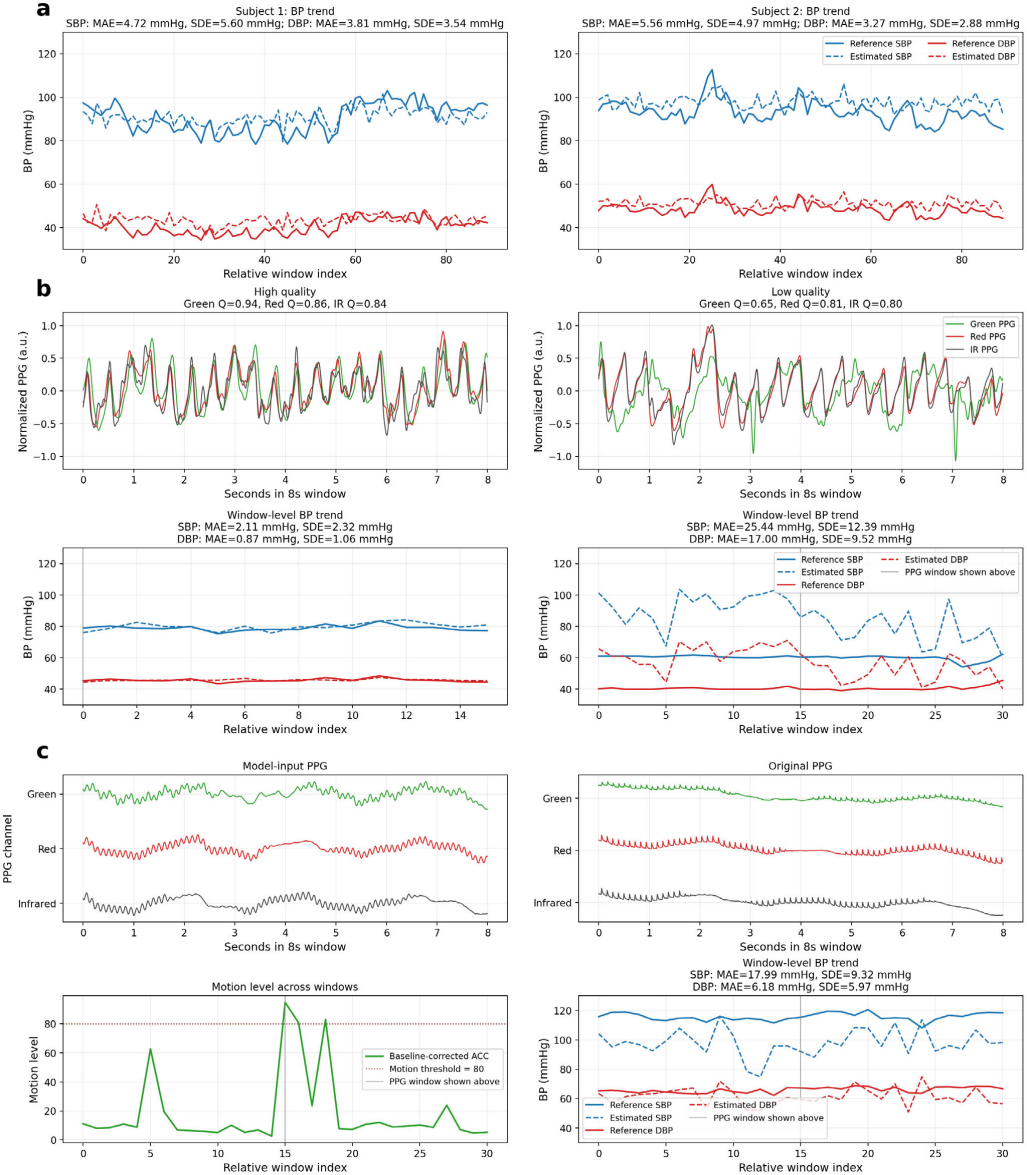
Representative examples of BP‐estimation performance across temporal trends, signal quality, and motion conditions. (a) Estimated vs. reference SBP/DBP over consecutive windows for two recordings. (b) High‐ and low‐quality 8‐s PPG examples (top) with corresponding local BP trends (bottom); gray markers indicate the position of the shown PPG window. (c) A motion‐contaminated example showing model‐input PPG, original PPG, and baseline‐corrected acceleration magnitude, shown for interpretation only and not used as model input, with the corresponding BP trend. *Note*. In all panels, solid and dashed lines denote reference and estimated BP, respectively; blue and red denote SBP and DBP, respectively. MAE and SDE denote the mean absolute error and standard deviation of error over the displayed segment. Green‐Q, Red‐Q, and IR‐Q denote channel‐specific SQI values of the selected window.

The subgroup analysis was conducted as a retrospective error‐stratification analysis of the trained G+R+IR model, rather than as a prospective confidence‐estimation module. In this study, error stratification refers to a descriptive subgroup error profile. Test segments were grouped by predefined acquisition‐related criteria in Table 3, including motion intensity and channel‐specific SQI, and MAE and SDE were compared across groups. For the IMU‐based motion‐intensity analysis, the higher‐ and lower‐motion‐intensity strata together cover all analyzed test segments in Table 3. We compared MAE and SDE across groups, but this analysis was not designed as a trend‐consistency test, correlation analysis, or significance‐tested endpoint. Estimation error varied across acquisition conditions (Figure 4d). Higher motion intensity was associated with larger SBP and DBP errors, although this subgroup should be interpreted cautiously because of its small sample size. Higher signal quality, stable intra‐window BP labels, normal‐range HR, and smaller $\Delta \mathrm{HR}/\Delta {\mathrm{SpO}}_{2}$ were generally associated with lower estimation errors. The NICU (Congenital CVD) subgroup showed lower MAE and SDE than the NICU (Non‐CVD) subgroup, but this observation should be interpreted descriptively. We consider that this phenomenon may be related to differences in signal quality, because the two subgroups also differed in PPG SQI: the congenital‐CVD subgroup showed higher average PPG SQI, particularly in the green channel, than the non‐CVD subgroup (Table 5). Therefore, the lower MAE and SDE in the congenital‐CVD subgroup are more conservatively interpreted as an acquisition‐quality‐associated observation in the current cohort. Whether congenital CVD itself contributes to this subgroup difference requires further investigation. The physiological influence of congenital cardiovascular disease status on this waveform‐based BP model remains unclear. Other factors, including motion exposure, supportive care, measurement‐site availability, and site/session distribution, were not prospectively balanced or included as covariates. Therefore, the CVD/non‐CVD comparison should not be interpreted as a causal effect of congenital cardiovascular diagnosis.

**TABLE 5:** Signal quality assessment under different scenarios.

Subgroup	Green SQI	Red SQI	Infrared SQI
Overall	0.79±0.11	0.80±0.08	0.81±0.08
No Ventilation	0.71±0.12	0.75±0.12	0.76±0.12
Conventional Ventilation	0.83±0.10	0.82±0.08	0.81±0.08
High‐frequency Ventilation	0.74±0.07	0.80±0.06	0.81±0.05
NICU (Non‐CVD)	0.76±0.11	0.80±0.08	0.79±0.08
NICU (Congenital CVD)	0.86±0.08	0.83±0.08	0.83±0.08

Intra‐window blood pressure stability was also associated with model performance. The subgroups with SBP STD ≤ 2 mmHg and DBP STD ≤ 2 mmHg showed relatively low error, indicating better performance when the reference BP labels were stable. This result highlights a limitation of using a single window‐averaged label during periods of rapid BP fluctuation.

Furthermore, the neonate's physiological state also influenced estimation accuracy. Regarding heart rate, model performance remained stable within the core neonatal range (HR 100–180 bpm), but the error increased markedly when the heart rate was very high (HR 180–300 bpm).

The analysis also investigated the discrepancies (Δ) between vital signs derived from the device's PPG signals and synchronized bedside‐monitor readouts. Here, ΔHR denotes the absolute difference between wearable PPG‐derived HR and the electrocardiogram (ECG)‐derived monitor HR readout, and $\Delta {\mathrm{SpO}}_{2}$ denotes the absolute difference between device‐derived and monitor‐derived ${\mathrm{SpO}}_{2}$. The results revealed that when these discrepancies were smaller (e.g., ΔHR≤ 8 bpm and $\Delta {\mathrm{SpO}}_{2}\leq$ 2%), the corresponding blood pressure estimation error was also lower. This association suggests that the agreement between these intermediate physiological measurements may serve as a retrospective proxy for the underlying signal quality and, consequently, for error‐prone acquisition states. However, ΔHR and $\Delta {\mathrm{SpO}}_{2}$ were analyzed retrospectively in this study and were not integrated into the BP regressor or an online confidence‐output branch. They are therefore treated as candidate variables for future confidence or error‐risk modeling, not as deployed dynamic confidence indicators.

In summary, this analysis clearly delineates the model's performance boundaries. While DBP estimation demonstrated stability across most clinical scenarios, SBP accuracy proved highly sensitive to specific acquisition states. Specifically, optimal monitoring accuracy is contingent upon low motion interference, high signal quality, stable intra‐window blood pressure, and consistent physiological baselines, such as a normal heart rate. Furthermore, high fidelity between device‐derived vitals and gold‐standard measurements may indicate more reliable acquisition conditions, but a calibrated per‐reading confidence score remains to be implemented and prospectively validated.

### 3.7 Heart Rate and Pulse Oximetry

The system also enables HR and ${\mathrm{SpO}}_{2}$ measurement. Heart rate estimation relies on the periodicity of the alternating current (AC) component of the PPG signal and derives instantaneous HR from the inter‐beat interval (IBI) between consecutive pulse wave peaks. Multi‐wavelength PPG (green, red, infrared) provides redundant support for robust estimation. An integrated dynamic signal quality assessment and channel selection mechanism calculates the signal quality index (SQI) for the three channels for each analysis window. It then performs peak detection and IBI estimation using the optimal channel or a weighted fusion, thereby maintaining the stability and precision of the heart rate output under interference. The algorithm workflow involves preprocessing the raw PPG with band‐pass filtering, followed by automatic multi‐scale peak detection to accurately locate pulse wave peaks and calculate HR [28]. Heart‐rate estimation results across wavelength combinations are summarized in Table 4.

The principle of ${\mathrm{SpO}}_{2}$ measurement relies on the differential absorption spectra of oxygenated hemoglobin ($HbO_{2}$) and deoxygenated hemoglobin (Hb) at red (640 nm) and infrared (950 nm) wavelengths. To construct the ratio‐of‐ratios parameter (R), the pulsatile (AC) and baseline (DC) components of the two signals are extracted:

$$R=\frac{AC_{RED}/DC_{RED}}{AC_{IR}/DC_{IR}}$$

where $AC_{RED}$ and $DC_{RED}$ are the AC and DC components of the red light (640 nm) PPG, and $AC_{IR}$ and $DC_{IR}$ are the corresponding components for infrared light (950 nm). Theoretically, there is a nonlinear relationship between R and ${\mathrm{SpO}}_{2}$. In practice, an empirical calibration curve is used to establish the mapping. This study utilized clinical data from postoperative patients covering a wide reference range. The device‐calculated R values were fitted against synchronized gold‐standard ${\mathrm{SpO}}_{2}$ from monitors using polynomial regression to obtain a calibration model that accurately maps R to ${\mathrm{SpO}}_{2}$. Following calibration, the system's ${\mathrm{SpO}}_{2}$ measurement achieved an MAE of 2.37% and an RE of 2.56%.

## 4 Discussion

This study addresses the critical reliability gap in neonatal cuffless blood pressure monitoring by performing a reliability‐oriented error analysis based on clinical data. Estimation error was heterogeneous across acquisition conditions, as shown by the descriptive subgroup profile based on MAE and SDE. This descriptive subgroup error profile supports quality‐aware data curation and identifies candidate variables for future confidence‐estimation models.

Figure 5 provides a visual link between the aggregate error profile and local waveform behavior. The representative examples illustrate that BP‐estimation errors may appear as local trend offsets, transient mismatches, or increased error dispersion when PPG quality is low or motion is present. This visualization helps readers interpret MAE and SDE at the local trend level, while remaining descriptive rather than a calibrated per‐reading confidence output.

A key enabling component for this evidence‐based analysis is multi‐wavelength PPG sensing. Different wavelengths exhibit distinct penetration depths and absorption characteristics in tissue, allowing the capture of hemodynamic information across superficial and longer optical paths. Prior wearable PPG studies support wavelength diversity as a strategy for motion‐artifact handling, physiological‐parameter estimation, and BP‐related inference. In particular, red/infrared measurements provide deeper optical penetration than green light [17], while multi‐wavelength PPG has been used to exploit wavelength‐dependent light penetration for BP‐related inference [29]. Consistent with this rationale, our internally controlled wavelength‐combination analysis showed that the G+R+IR input achieved the lowest SBP MAE, DBP MAE, and SBP/DBP SDE among the tested configurations. We interpret this ablation as functional evidence that wavelength‐specific and geometry‐specific PPG channels provide complementary BP‐related waveform information in the present neonatal dataset. This conclusion is limited to the current neonatal NICU acquisition setting and the controlled wavelength‐combination ablation. This finding is consistent with the broader MWPPG rationale that wavelength diversity can provide complementary pulsatile information, but we do not interpret it as a direct head‐to‐head superiority claim over existing MWPPG‐based BP methods because prior studies differ in population, optical geometry, reference BP, calibration assumptions, and auxiliary inputs. Instead, the present result supports MWPPG as a practical signal‐level strategy in our neonatal NICU acquisition setting.

Building on this sensing foundation, our findings provide quantitative criteria for defining data segments that support robust model performance. Traditional approaches often rely on uniform band‐pass filtering, which does not fully address the heterogeneity of clinical data quality. By contrast, prior MWPPG‐based methods have mainly pursued signal‐level strategies for motion handling. Liu et al. [20] applied a PCA‐based decomposition to separate arterial and capillary pulse components in elderly subjects and used a PCA‐derived motion component to detect and exclude motion‐contaminated segments before pulse‐transit‐time‐based BP estimation. Hou et al. [21] used camera‐based, contact‐free MWPPG in adults and a deep‐learning model to learn wavelength‐dependent morphological features directly from green, red, and near‐infrared waveforms for BP regression, without an explicit motion‐ or quality‐based stratification step. In contrast, our subgroup analysis establishes a descriptive error‐stratification profile that characterizes, rather than actively corrects, how estimation accuracy varies systematically with intrinsic PPG quality metrics (SQI) [30], motion intensity, clinical subgroup, and the within‐window variability of invasive ABP labels.

Anatomical placement and repeated sessions were not prospectively balanced, and the global chronological split was not grouped by neonate. Therefore, the reported results should be interpreted as within‐cohort segment‐level evidence for wavelength‐combination comparison and quality‐aware error characterization, rather than as subject‐independent validation on completely unseen neonates. This evidence supports a shift in emphasis from signal processing alone toward data curation. In this framework, quality‐aware screening based on error‐predictability markers may help models learn stable mappings from physiological signals to BP while reducing the influence of artifacts, confounders, and label instability.

These quality‐error associations support quality‐aware interpretation of segment‐level BP estimates and identify SQI and motion intensity as candidate variables for future dynamic confidence metrics. However, this study did not train or validate an online per‐reading confidence model. To provide a balanced reference for accuracy evaluation, we summarized BHS/ISO/IEEE‐related metrics, including ME, SDE, MAE, and CPE, as contextual references for interpreting the reported SBP/DBP errors [31, 32, 33, 34]. These metrics are provided only as contextual performance references and are not used to claim formal BHS/ISO/IEEE validation, grading, compliance, or device certification. In our study, DBP estimation exhibited lower dispersion under high‐quality conditions, suggesting that DBP estimates maintain higher absolute accuracy in favorable scenarios. By contrast, SBP remained more sensitive to environmental and physiological perturbations and may be more appropriate for trend monitoring, consistent with broader observations in cuffless BP research [16]. These results could inform future systems that report a numerical BP value together with an interpretable confidence metric, thereby supporting safer integration into clinical workflows.

The subgroup analysis suggests a stepwise roadmap for clinical translation. Under scenarios with reduced motion and improved coupling, estimation errors decrease substantially and move toward clinically relevant ranges under constrained conditions. These results support constrained‐use scenarios with reduced motion, while broader artifact‐robust deployment requires larger, balanced validation.

Beyond algorithm performance, the wireless system may improve neonatal care workflows. It can reduce wired clutter, lower the risk of skin injury associated with repeated adhesives, and remove barriers to parent‐infant interaction, including kangaroo mother care. Reliable continuous data may also reduce disturbances from repeated spot checks, which is particularly valuable for postoperative or mechanically ventilated neonates who require positional stability.

Several limitations should be acknowledged. First, the cohort size was limited, and generalizability requires larger multicenter validation. The primary evaluation used a global chronological segment‐level split to provide a stable benchmark for a NICU dataset with limited size and heterogeneous recording durations. This design preserves temporal structure and mitigates segment imbalance from unequal recording lengths. However, because the split was based on pooled time‐ordered segments rather than subjects, segments from the same neonate could appear in multiple partitions. The reported results therefore reflect within‐cohort chronological segment‐level performance, not subject‐independent generalization. This overlap may introduce subject‐specific information and implicit calibration that improves observed performance. Larger multicenter validation is needed across unseen subjects, anatomical sites, clinical conditions, and centers.

Second, the observed quality–error associations were derived from one dataset and one primary neural‐network backbone. They may therefore be influenced by model architecture, training split, site distribution, and clinical subgroup composition. Time‐of‐day and circadian effects were not explicitly modeled or evaluated in this study; therefore, potential circadian influences on BP labels, PPG characteristics, and estimation errors cannot be excluded. Future work will incorporate circadian information as a model input, which may improve system robustness in real‐world clinical settings.

Third, the current BP regressor was designed as a waveform‐only MWPPG estimator. SQI, IMU‐derived motion, $\Delta \mathrm{HR}/\Delta SpO_{2}$, intra‐window BP STD, and skin‐interface temperature were used for retrospective reliability characterization rather than as auxiliary inputs or gating signals. Direct integration of these variables would require a separate multimodal or gated model with dedicated ablation validation, especially because intra‐window BP STD is ABP‐label‐derived and $\Delta \mathrm{HR}/\Delta SpO_{2}$ depend on bedside‐monitor readouts. Wearable‐derived SQI and IMU motion are practical candidates for future auxiliary‐input, adaptive‐weighting, or confidence‐gating models. These variables should therefore be considered candidates for future reliability modeling, not validated contributors to the current BP estimator.

Fourth, temporal mismatch between PPG and ABP was evaluated using simulated ±1–5 s time shifts. The resulting SBP/DBP label drift was limited, reaching 1.0/0.7 mmHg at ±1 s and 2.2/1.6 mmHg at ±5 s. These values were lower than the overall test‐set MAE of 9.67/6.17 mmHg. This limited drift is consistent with the segment‐level ABP averaging used for fixed‐window PPG‐BP labels [35]. Because the current study uses 8‐s segment‐level labels, this analysis should be interpreted as label‐level sensitivity for segment‐level BP estimation. Finer‐grained tasks, such as waveform‐level PPG‐to‐ABP reconstruction, require stricter PPG‐ABP synchronization because they are more sensitive to sensor‐clock asynchrony, system delays, device placement variability, and firmware or driver latency [36].

Fifth, the current BP estimator predicts segment‐level BP rather than instantaneous or beat‐level BP. The use of mean 1 Hz ABP‐derived SBP/DBP values within each 8‐s window improves label stability but may smooth rapid BP fluctuations. Beat‐level BP estimation is therefore a valuable future direction because it could capture beat‐to‐beat hemodynamic changes that are not represented by window‐averaged labels. Achieving this goal requires coordinated advances in hardware, software, and physiological modeling. Hardware improvements are needed to provide more precise synchronization, stable sensor‐tissue coupling, and low‐latency acquisition. Software development should include robust beat detection, beat‐level signal quality control, adaptive temporal alignment, and uncertainty estimation. Mechanistically, future studies should further characterize how neonatal PPG morphology, vascular compliance, peripheral perfusion, and sensor placement jointly affect beat‐to‐beat BP dynamics.

In summary, this study characterizes reliability‐related determinants of neonatal cuffless BP estimation rather than optimizing a BP estimator in isolation. By analyzing determinants of cuffless BP accuracy in a real NICU environment, the results support quality‐aware screening and motivate systems that can eventually report calibrated per‐reading confidence information alongside BP estimates. Together with workflow benefits from the wearable system, this reliability‐aware pathway supports stepwise translational evaluation in neonatal care.

## 5 Experimental Section

### 5.1 Hardware Design Rationale and Component Selection

The NCBP band was engineered around a custom fPCB to meet the primary design goal of conforming to neonatal limbs. A flexible ribbon structure connects the main circuit areas (Figure 1c), providing mechanical extensibility for a secure yet comfortable fit that supports stable signal acquisition across neonatal sizes.

The system core is a Silicon Labs EFR32BG22 SoC, selected for its energy‐efficient ARM Cortex‐M33 architecture and integrated Bluetooth 5.2 stack. This single‐chip solution manages sensor orchestration, on‐device preprocessing, and wireless transmission while adhering to the strict power constraints of continuous neonatal monitoring. Synchronous acquisition from the optical and inertial front‐ends is maintained via a high‐speed Serial Peripheral Interface (SPI) bus, ensuring temporal alignment across all data streams.

A multi‐wavelength PPG unit integrates green (570 nm), red (640 nm), and infrared (950 nm) illumination. The optical layout uses a reflective green channel and transmissive red/infrared channels to enable concurrent heart rate extraction, ${\mathrm{SpO}}_{2}$ derivation, and complementary BP‐related waveform acquisition. The same temporal TDM control is applied to all wavelengths, whereas the spatial optical geometry differs by wavelength. The green channel uses a short‐path reflective configuration for relatively motion‐robust superficial pulse tracking [29], while the red/infrared channels use a longer‐path transmissive configuration to preserve the oximetry pair and provide complementary deeper‐path PPG waveform inputs for BP‐related inference [18, 37]. A dedicated analog front‐end (Texas Instruments AFE4410) drives LEDs, conditions the signal, and digitizes the photodiode output.

To provide contextual data for acquisition monitoring and ensure safety, the system incorporates an STMicroelectronics LIS2DW12 tri‐axial accelerometer to monitor motion status and multiple temperature sensors to track the skin‐interface temperature. In the present analysis, skin‐interface temperature measurements were used only for safety and acquisition‐context monitoring and were not included as inputs to the BP regression model or as variables in the retrospective error‐stratification analysis. The power management architecture was designed for reliability and safety, utilizing dedicated integrated circuits for intelligent charge/discharge path management (CWI CW6307) and a stable operating voltage supply (ETA‐SEM ETA5071). For the engineering power estimate, LED contributions were calculated from the optical TDM duty cycle by assuming 20–30 mA current pulses and 60–100 μs on‐time per wavelength slot at 100 Hz, while MCU/BLE, AFE receiver, accelerometer, temperature‐sensing, and power‐management contributions were estimated from vendor datasheet current values under continuous streaming. This yielded an average‐current estimate of approximately 2.5–3.6 mA and a transient peak‐current estimate of approximately 25–40 mA.

### 5.2 Device Fabrication and Encapsulation

The device's fabrication and encapsulation were guided by the clinical requirements of biocompatibility, comfort, and optical isolation. Following the assembly of all components onto the fPCB, the entire circuit was encapsulated within a soft, medical‐grade silicone shell (Shore A hardness of 30) chosen for direct contact with fragile neonatal skin. To mitigate ambient light interference, the silicone enclosure was rendered with a high‐opacity black pigment to suppress external light intrusion and reduce internal light leakage. The final form factor was achieved using injection molding, and the populated fPCB was sealed within the preformed structures using a transparent, biocompatible adhesive at the optical windows to protect the electronics from moisture ingress and mechanical stress. The finished device measures approximately 130 × 20 × 8 mm and weighs 8.3 g.

### 5.3 Clinical Application Observations and Usage Considerations

Initial clinical use showed that the flexible structure and skin‐friendly materials conform to the wrist, ankle, and foot. This conformity supports stable optical‐mechanical coupling, which is required for high‐quality signal acquisition. The adjustable hook‐and ‐loop fastener adapts quickly to different neonates. Sites not occupied by clinical probes should be preferred to avoid interference. The medical‐grade silicone showed good biocompatibility, with no irritation observed after continuous use. The sealed design also supports surface disinfection by wiping or spraying with medical alcohol, such as 75% ethanol.

In clinical practice, device tightness is a key determinant of signal quality. A loose interface can cause ambient light leakage and optical‐path attenuation, reducing signal‐to‐noise ratio. Moderate pressure improves waveform fidelity, whereas excessive compression can impair local perfusion and cause PPG distortion or signal loss. Optimal placement also depends on optical modality. The reflective green channel requires alignment with target vasculature on the same skin surface. The transmissive red and infrared channels are less sensitive to rotation but require a sufficiently thin anatomical site for light penetration. Poor peripheral perfusion often requires adjustment of sensor location and strap tension. These observations support standardized procedures for neonatal wearability, safety, hygiene, and signal acquisition.

### 5.4 Data Recording and Signal Processing

The embedded firmware communicates with the AFE and the inertial measurement unit (IMU) via SPI. Multi‐wavelength PPG signals are sampled at 100 Hz per optical channel, while IMU data are acquired at 25 Hz. The 100 Hz rate corresponds to a Nyquist frequency of 50 Hz, which covers the 0.4–10 Hz PPG analysis passband used in this study and the neonatal pulse‐rate range of interest, including the 180–300 bpm high‐HR subgroup. At 300 bpm, one cardiac cycle lasts about 200 ms and yields roughly 20 PPG samples at 100 Hz. Although a higher rate such as 400 Hz (80 samples per beat) is beneficial for fine beat‐level timing or derivative‐based morphology, the present model estimates short‐window averaged BP from 8‐s PPG segments. Thus, 100 Hz provides sufficient temporal resolution for segment‐level waveform morphology analysis. The 25 Hz IMU stream is used to quantify segment‐level motion intensity for retrospective error stratification.

For on‐device preprocessing, the firmware subtracts the ambient‐light PD readout acquired during the LED‐off phase from each LED‐on measurement. The corrected green, red, and infrared PPG samples are then stored or transmitted. Consequently, the BP regression model receives ambient‐corrected PPG waveforms rather than a separate ambient‐light channel. This design serves acquisition‐level artifact suppression and was not intended to encode additional BP‐related physiological information.

Reference readouts are obtained from a bedside multi‐parameter monitor at 1 Hz, including SBP, DBP, MAP, ECG‐derived heart rate (HR), and ${\mathrm{SpO}}_{2}$. The ECG‐derived HR shares the same 1‐s timestamp axis as the ABP‐derived BP readouts and is used only as an auxiliary reference for retrospective ΔHR analysis; raw ECG waveforms were not exported or used as model inputs. ABP‐derived SBP and DBP served as the gold‐standard BP references for model training, validation, and testing.

Both the ABP monitor and the wearable PPG signals were aligned to the local hospital standard time. ABP‐derived monitor readouts carried 1‐second‐resolution reference timestamps, and PPG data were timestamped on the same time base, keeping the timing error between the two signals within 1 second. Across the analyzed recordings, the valid multi‐wavelength PPG data had a total duration of 110.66 h. At the subject level, valid recordings from the same neonate were aggregated before calculating the duration statistics. The valid recording duration ranged from 1.393 to 5.077 h per subject, with an average duration of 2.634 h. Recording timestamps were used exclusively for alignment and chronological partitioning; they were neither supplied to the BP regressor nor employed to construct day/night strata.

All acquired physiological signals and their synchronized reference labels are transmitted wirelessly in real time to a base station or mobile terminal via Bluetooth. Data streams are visualized on both desktop and mobile platforms and are stored in a de‐identified format to mitigate privacy risks while preserving temporal alignment for subsequent analysis.

Continuous PPG signals were segmented into 8 s windows with a 2 s step (6 s overlap). For each segment, SBP and DBP targets were defined as the mean of the synchronized 1 Hz invasive‐ABP‐derived readouts within the same 8 s interval. This approach reduces the influence of transient outliers but defines the prediction target as short‐window averaged SBP/DBP. The model should therefore be interpreted as a segment‐level BP estimator. Because training used segment‐level labels rather than beat‐level ABP waveform targets, the approach reduces dependence on sub‐second phase matching but may smooth rapid BP changes. A fourth‐order Butterworth bandpass filter with a 0.4 to 10 Hz passband was applied to reduce baseline drift and high‐frequency noise. Segment amplitudes were z‐score normalized to reduce inter‐subject scale differences. SQI was computed for each PPG segment following established metrics [30]. The BP regressor uses only the normalized PPG waveform segments as input. SQI, IMU‐derived motion measures, $\Delta \mathrm{HR}/\Delta {\mathrm{SpO}}_{2}$, and intra‐window SBP/DBP STD are not provided to the regression network. These variables are reserved for retrospective reliability analysis.

Because enrolled neonates were receiving routine NICU care, recordings could be interrupted by clinical procedures, supportive therapies, or nursing workflows. Recording lengths and segment counts therefore varied across neonates and sessions. Anatomical‐site availability and clinical‐state distributions were also governed by bedside feasibility rather than balanced sampling. All eligible segments were pooled, sorted chronologically by recording timestamp, and split into training (60%), validation (20%), and testing (20%) sets. This global chronological split was used as the primary segment‐level benchmark to preserve the naturally observed clinical acquisition distribution. Earlier, middle, and later time‐ordered segments were assigned to the training, validation, and testing sets, respectively. A mean baseline was additionally included as a waveform‐free reference, using the training‐set mean SBP and DBP labels as constant predictions for all held‐out test segments and evaluating them with the same error metrics as the neural‐network backbones. Because the split operated on the pooled segment sequence rather than on subjects, segments from the same neonate could appear in more than one partition. The reported results therefore represent within‐cohort chronological segment‐level performance rather than strict subject‐independent generalization.

The core regressor employs a 1D‐EfficientNet encoder, selected as a state‐of‐the‐art open‐source backbone for physiological time‐series analysis. This encoder is composed of MBConv1D modules and followed by a lightweight regression head that outputs SBP and DBP [38]. To improve representation transfer, the encoder is initialized from a foundation model pre‐trained on large‐scale wearable biosignals using contrastive self‐supervised learning, and the entire network is then fine‐tuned end‐to‐end on the neonatal dataset [39]. This strategy is consistent with recent efforts in self‐supervised and masked‐autoencoder‐based biosignal representation learning [40]. The model was trained with Huber loss for robustness to outliers and optimized with Adam. After training, the saved model was evaluated on the held‐out segment‐level test set. Additional waveform‐based deep‐learning backbones were evaluated with the same split, preprocessing pipeline, and SBP/DBP targets. Computational efficiency was profiled using MACs, parameter count, per‐segment latency, throughput, serialized model size, memory footprint, processor‐side power, and energy per sample. These metrics characterize neural‐network inference under the benchmark environment.

### 5.5 Study Cohort and Clinical Acquisition Environment

This study included 42 NICU neonates who were already undergoing invasive ABP monitoring as part of routine clinical care. The cohort exhibited broad demographic variability, with a mean gestational age of 36.8 ± 4.2 weeks and a mean birth weight of 2850 ± 1150 g. Primary NICU admission indications included prematurity‐related complications, neonatal respiratory distress syndrome, and suspected sepsis. For clinical‐status subgroup analysis, neonates with congenital cardiovascular disease were assigned to the congenital‐CVD subgroup, and neonates admitted for non‐cardiovascular critical conditions were assigned to the non‐CVD subgroup. This grouping was clinically relevant because hemodynamic status, monitoring indications, supportive care, motion exposure, and measurement‐site constraints may differ between groups. It was not designed as a diagnosis‐matched comparison or as a test of the causal effect of cardiovascular diagnosis.

Data were collected under real‐world NICU conditions. Long‐term continuous wearable monitoring is inevitably affected by clinical care and supportive therapies, which may introduce heterogeneous signal quality (e.g., motion‐related artifacts under spontaneous limb activity, or vibration‐induced interference under high‐frequency ventilation). To characterize these acquisition conditions, segment‐level PPG SQI was computed using established metrics [30] and used to support downstream analyzes of model robustness across clinical scenarios (see Table 5).

The multi‐site measurement strategy was adopted because wearable recordings were obtained during routine NICU treatment without interfering with existing clinical care. For neonates without major cardiovascular diseases, such as aortic coarctation, prior four‐limb blood pressure studies suggest that large limb pressure gradients are uncommon [41, 42]. This premise was not used to assume equivalence across PPG acquisition sites. Trained clinical staff selected one of eight predefined distal sites ‐ left/right palm, left/right wrist, left/right ankle, and left/right foot ‐ based on clinical feasibility and instantaneous signal quality. Sites affected by arterial lines, pulse‐oximetry probes, intravenous access, skin injury, edema, poor perfusion, or ongoing nursing procedures were avoided when possible. Measurement site can influence PPG quality through tissue thickness, perfusion, motion exposure, and sensor‐skin coupling. These factors alter optical path, pulsatile amplitude, and waveform morphology, which can affect BP‐regression error. Site‐related BP‐estimation differences should therefore be interpreted as effects of acquisition quality rather than differences in systemic BP. Because site availability and sample counts were not prospectively balanced, the site analysis should be viewed as a feasibility assessment with exploratory observations rather than a definitive location‐level reliability ranking. In practice, probe placement should be guided by instantaneous PPG quality, stable coupling, adequate perfusion, skin condition, and avoidance of competing clinical probes.

As the reference standard, all neonates were monitored with a bedside multi‐parameter monitor. The monitor acquired invasive ABP at 500 Hz through an arterial catheter and reported derived vital‐sign readouts, including SBP, DBP, MAP, HR, and ${\mathrm{SpO}}_{2}$, at 1 s resolution. Wearable streams were synchronized to the monitor timeline using unified timestamps, and ABP‐derived SBP/DBP readouts served as training targets for segment‐level analyzes.

### 5.6 Ethical Approval and Experimental Design for Human Study

This study was conducted in accordance with the Declaration of Helsinki and approved by the Ethics Committee of Shanghai Children's Medical Center, School of Medicine, Shanghai Jiao Tong University (Approval No. SCMCIRB‐K2024014‐1) and the Ethics Committee of Fujian Children's Hospital (Fujian Branch of Shanghai Children's Medical Center) (Approval No. 2023ETKLRK11001). All enrolled neonates were already undergoing invasive arterial blood pressure monitoring as part of routine clinical care; no additional invasive procedures were performed for research purposes. Before participation, written informed consent was obtained from the parents or legal guardians of all neonates after they had been informed of the study purpose, procedures, and the use of de‐identified clinical data. All data were used exclusively for research on neonatal physiological monitoring and noninvasive blood pressure estimation. To ensure confidentiality, all personal identifiers were removed prior to data storage, analysis, and presentation.

## Author Contributions

W.S., M.W., and Z.D. designed the system. L.M. contributed to the conceptualization, obtained the multicenter ethical approvals, and conducted the safety and stability testing with Z.D. L.M., B.L., and Y.L. conducted the clinical studies, patient recruitment and follow‐up. J.C., W.S., and B.L. performed the analyzes and had access to original data. W.S., J.C., and R.G. drafted the manuscript. C.C., G.W., W.G., and W.L. provided expertise and supervision throughout the study. All authors have read and approved the manuscript.

## Conflicts of Interest

The authors declare no conflicts of interest.

## Data Availability

The data that support the findings of this study are available from the corresponding author upon reasonable request, subject to institutional approval, ethical requirements, and applicable data‐use agreements.
